# A survey of the practice and experience of clinical educators in UK secondary care

**DOI:** 10.1186/1472-6920-14-229

**Published:** 2014-10-23

**Authors:** Robert I Norman, Nisha Dogra

**Affiliations:** Department of Medical and Social Care Education, University of Leicester, Leicester, LE1 9HN UK; School of Psychology, University of Leicester, Leicester, LE1 9HN UK

**Keywords:** Medical students, Trainees, Clinical educators, Secondary care, Trusts, Deanery, Attitudes, Experience

## Abstract

**Background:**

Experiences and attitudes of clinical trainers of undergraduate medical students and postgraduate medical trainees in secondary care have received limited attention. Anecdotally, clinical teaching is becoming increasingly restricted by clinical service pressures, thereby presenting a risk to the quality of training provision.

**Methods:**

To explore the commitment, experience and attitudes of clinical teachers and trainers of undergraduate medical students and postgraduate trainees, respectively, amongst secondary care providers across a UK Healthcare Workforce Deanery, an invitation to complete a study-specific, on-line survey, comprising predominantly yes/no response and 5-point Likert scale statements with some open questions, was sent to all registered secondary care trainers/supervisors working in the East Midlands Strategic Health Authority. The survey was open between February and June 2012, with two reminders to complete. Responses were anonymised and the frequency of responses to questions was analysed. Data were analysed for the whole study population and for the relationship between frequency of responses and gender.

**Results:**

The majority of teachers/trainers considered that they were well prepared and fulfilled their clinical teaching responsibilities. Many reported having restricted time for preparation and delivery and that teaching activities were often completed in their own time. Despite reported poor support and low incentives, many respondents felt valued for their clinical teaching by their Medical Schools and the Deanery, but less so by hospital Trusts.

**Conclusions:**

Respondents indicated that some faculty like and enjoy clinical teaching despite lack of allocated time, resources and recognition. The majority indicated that they feel confident and competent in their clinical teaching roles. Insufficient dedicated time due to competing clinical service pressures was reported as the major barrier to clinical teaching provision.

## Background

Clinical education is provided predominantly by non-faculty appointed clinical teachers (undergraduate) and trainers (postgraduate) most of whom undertake this work as a part of their clinical service contract, primarily as clinical practitioners. Whilst there is an administrative divide between medical schools and deaneries for undergraduate and postgraduate clinical education, respectively, clinical teaching and training is often provided by the same people to the different levels and in the context of busy clinical service demands.

The understanding between medical schools and postgraduate deaneries and partner healthcare providers is that their relationship is mutually beneficial but increasing pressures in all domains to improve efficiency and quality in their respective activities places a dual strain on clinical educators. Additional pressures arise from the regulatory requirements in the UK of the General Medical Council against ‘Tomorrow’s Doctors (2009)’ [1] and ‘The Trainee Doctor’ (2011) [2] for undergraduate and postgraduate training, respectively. Given that education responsibilities generally represent only a fraction of contracted duties, clinical educators are under considerable pressure to balance contracted teaching time with the demands of their clinical service and other responsibilities. Surprisingly, there is little research to assess the impact of these pressures on the delivery of clinical education.

Evaluations of students’ experiences of medical teaching have been made at both undergraduate [3–8] and postgraduate [9] levels. In contrast, only limited attention has been given to the experiences of teachers of medical undergraduates [10, 11] and almost none of teacher/trainers at the postgraduate clinical level [12]. An evidence base is required against which to structure future teaching provision and support. Despite the paucity of data on clinical teacher experiences and attitudes, some common themes have emerged already from the available studies of undergraduate teaching. While teachers report their enjoyment of teaching in general, both Seabrook [10] and Hendry et al. [11] reported a potential disjunction between contractual expectations and the perceived teaching experience of teachers. Significant issues raised by both studies included, workload pressures that resulted in teaching time being notional rather than protected and lack of time for preparation, poor availability of appropriate space and financial resources for teaching materials. Also highlighted was poor communication between those commissioning training with staff resulting in staff feelings of isolation, poor preparation of students for clinical practice and lack of institutional recognition and potential reward. A qualitative study of 26 clinical academic teachers of undergraduate psychiatry over 23 different medical schools revealed similar themes of conflict of time between service and teaching, lack of resourcing, support and status [13].

Given that themes emerging from studies on undergraduate teachers are likely to be duplicated for postgraduate clinical trainers, there are potentially a number of areas where significant improvement in training provision should be made. The aim of this study was to survey teachers and trainers of undergraduate medical students and postgraduate trainees, respectively, across secondary care providers in the East Midlands Strategic Health Authority (EMSHA), to determine the time commitment and level of engagement of clinical trainers in both undergraduate and postgraduate clinical education, to establish the level of training in clinical education received by clinical educators and to ascertain trainer experiences, attitudes and barriers to their clinical education responsibilities, to support quality improvement.

## Methods

A study specific on-line survey was constructed by consensus of investigators (RIN, ND) using elements from published validated surveys of undergraduate clinical educators [10, 11, 13] and postgraduate trainers [13]. The draft survey was piloted on three local faculty and three members of the EMSHA Quality Assurance Office for clarity, content and length and minor revisions made to reduce the number of questions. Approval for the study was obtained from the University of Leicester Research Ethics Committee. E-mail invitations to participate in the survey were sent via the EMSHA Quality Office to all (n = 2007) registered secondary care trainers/supervisors working in the EMSHA. The survey was delivered through the Bristol Online Surveys website (http://www.survey.bris.ac.uk) through a secure web link between February and June 2012, with e-mail reminders on two occasions in April and June. The survey was closed one month after the final reminder. Participants were assured that submissions of responses were anonymised to prevent attribution to individuals. The questionnaire was divided into seven sections: (1) ‘Demographic details’; (2) ‘Educational role description’; (3) ‘Teaching/training commitments’; (4) ‘Training for teaching’; (5) ‘Teaching Practice’ (6) ‘Experience of teaching’; (7) ‘Perceived value as a teacher/trainer’. The survey comprised predominantly of questions requiring either a yes/no response or 5-point Likert scale statements, with some open questions. For Likert items, participants were asked to respond to each statement with either ‘strongly agree’, ‘agree’, ‘neither agree nor disagree’, ‘disagree’ and ‘strongly disagree’, according to how much they supported the statement.

Responses were anonymised and the frequency of responses to questions was analysed and reported as ‘number of responses’ and ‘the percentage of the total number of responses’ for each question. Data were analysed for the whole study population, after duplicate survey submissions had been removed, and by gender. Correlations were only sought when sample sizes were large enough to allow meaningful analysis. Statistical analysis was performed using IBM SPSS Statistics version 20 (IBM Corporation, New York, USA). The chi-squared test was used to explore the relationship between frequency of responses and gender only, as most other groups were too small or disparate to enable meaningful comparisons. A p-value of <0.05 was considered significant.

## Results

### Teacher/trainer characteristics

Response rate declined to zero in the period before survey closure indicating saturation of potential responses. The survey was completed by 518 participants from 2007 registered secondary care trainers/supervisors surveyed (response rate of 25.8%). Duplicate submissions made by 34 participants were discarded before analysis. Respondent characteristics are shown in Table 1. Approximately two thirds of respondents were male. There was little gender difference in the responses with the exception that female respondents were significantly nearly twice as likely to have obtained formal teaching and or medical education qualifications at certificate or diploma level (Male, 9.0%; Female, 16.2%, p <0.02) but proportions progressing to a higher degree were not statistically different (Male, 7.5%; Female, 3.8%). Although numbers reporting a lack of confidence as a clinical educator were low, females were three times more likely than males to indicate a lack of confidence in this role (Male, 4.2%, Female, 13.0%, p <0.001), while there was no difference between the genders regarding perceived lack of competence as clinical educators (Male, 3.0%, Female, 3.2%).

**Table 1:** **Respondent demographics**

		n	%
Sex			
	Male	333	64.3
	Female	185	35.7
Age			
	Up to 35 years	16	3.1
	36-45 years	194	37.5
	46-55 years	241	46.5
	56-65 years	67	12.9
	66 years or above	0	0
Role description			
	NHS Consultant	467	90.2
	University Clinical Professor	7	1.4
	University Clinical Senior Lecturer	21	4.1
	Associate Specialist	13	2.5
	Other Specialty doctor	8	1.5
	Consultant level in non-NHS organisation	2	0.4
	GP	12	2.3
	Other	15	2.9
Education responsibility			
	Education Supervisor	398	76.8
	Clinical Supervisor	468	90.3
	College Tutor	42	8.1
	Training Programme Director	33	6.4
	Head of School	9	1.7
	Foundation Programme Director	5	1
	Clinical sub-dean	1	0.2
	Director of Medical Education/Clinical Tutor	6	1.2
	Deanery Associate Postgraduate Dean	4	0.8
	College Regional Adviser	18	3.5
	Other	74	14.3
Educational activities with			
	Undergraduate medical students	404	78
	FY1 trainees	310	59.8
	FY2 trainees	365	70.5
	CT level registrars	339	65.4
	ST level registrars	452	87.3
	GP registrars	155	29.9
	None at present	3	0.6

CT, Core Trainee; FY1/2, Foundation Doctor Year 1 or 2; GP, General Practitioner; ST, Speciality Trainee. nâ€‰=â€‰number of respondents.

The majority of respondents classified themselves as National Health Service (NHS) Consultant (Table 1). Numbers giving other designations and those indicating senior education roles were low and so analysis by these variables was not carried out. Responses were received from trainers in the majority of the 62 possible specialities listed in the survey (not shown). No speciality exceeded 16.0% of responses, so analysis by speciality was not carried out.

### Formal training for clinical education

The majority of respondents reported completion of continuing professional development training in ‘Roles of clinical and educational supervisors’ and ‘Work-based assessments and giving feedback’ (Table 2). Approximately two thirds of respondents reported completion of training in ‘Principles of training and learning’ and ‘Appraisal skills’ (Table 2) but fewer than half of respondents had received training in ‘Trainees in difficulty’. Most of the training received for educational roles related to postgraduate teaching and was delivered by the individual hospital Trusts and the Deanery, with lower levels of training received from external sources, predominantly the Royal Colleges and Speciality Associations (Table 3). Lower levels of training by the University Medical Schools for undergraduate teaching were also reported in the study population (Table 3). Approximately a quarter of respondents reported having some form of formal teaching qualification ranging from Certificate to PhD in Medical Education (Table 4). Of these, relatively few had progressed to a higher degree.

**Table 2:** **Continuing professional development education training received**

	Yes	No	% yes
Roles of Clinical & Educational Supervisors	469	49	90.5
Principles of Training & Learning	353	165	68.1
Appraisal skills	349	169	67.4
Work-based assessment & giving feedback	424	94	81.9
Trainees in difficulty	246	272	47.5
None at present	16		3.1

**Table 3:** **Sources of educational training**

	Number	% of respondents
Hospital Trusts	220	42.5
Deanery	296	57.1
University	75	14.5
Other, e.g. College or Speciality associations	156	30.1

**Table 4:** **Qualifications in medical education**

	Number	% of total participants	% of respondents
Certificate	43	8.3	30.9
Diploma	17	3.3	12.2
Masters	22	4.3	15.8
PhD	7	1.4	5.0
Other	50	9.7	36

### Clinical education responsibilities

Concerning educational activities with students and postgraduate trainees (Table 1), approximately one fifth of respondents had educational responsibility for postgraduate education at any level only. The remaining four fifths of respondents had undergraduate and postgraduate education roles and only five respondents reported having educational responsibility for undergraduates only. Three respondents reported having no current educational activity.

Twelve percent of respondents reported education activity with all six levels of trainee (Undergraduate (UG), Foundation Years 1 and 2 (FY1, FY2), Core Trainee (CT), Speciality Trainee (ST) and General Practice (GP) (Table 1). Approximately three quarters of respondents reported educational activity with FY1 and/or FY2 trainees and senior trainees at CT, ST or GP levels, while a quarter of respondents reported training activities with more senior trainees (CT, ST, GP) only (Table 1). Approximately half of respondents had educational responsibility for FY1 and FY2 trainees, while lower proportions reported training activity with FY1 or FY2 trainees only (Table 1).

### Teaching/training commitments

#### Undergraduate

A third of respondents reported a set number of hours per week in their job plan to teach undergraduate medical students (Table 5). Of these, the highest proportion had 1–3 hours per week allocated to undergraduate teaching. Approximately a quarter of respondents reported an allocation of less than 1 hour per week and a further quarter an allocation of 3 or more hours per week (Table 5). A greater proportion of respondents indicated that undergraduate teaching formed part of their Supporting Professional Activity (SPA) time allocation (Table 5). Of these, a third had no specific time allocation and a quarter was not sure. Only a small proportion reported an allocation within their SPA of one hour or more. Two thirds of respondents reported allocations of between one and three undergraduate medical students, with few being allocated more than six (Table 6).

**Table 5:** **Teaching/training commitments**

	Yes (%)	No (%)	Hours per week (%)				
			None	<1.0	1 to 3	>3.0	Not sure
Undergraduate/medical student teaching							
Set hours in job plan to teach/supervise medical students	169 (32.6)	349 (67.4)	0 (0)	46 (27.2)	76 (45.0)	39 (23.1)	8 (4.7)
Undergraduate teaching hours subsumed within SPA allocation	216 (41.7)	302 (58.3)	79 (35.6)	58 (26.9)	10 (4.6)	15 (6.9)	54 (25.0)
Spend more than allocated hours teaching UG students	200 (38.6)	318 (61.4)		34 (17.0)	120 (60.0)	17 (8.5)	29 (14.5)
Postgraduate teaching/supervision							
Set hours in job plan for postgraduate teaching/supervision	202 (39.0)	316 (61.0)	55 (27.2)	113 (55.9)	24 (11.9)	10 (5.0)	0 (0)
Postgraduate training hours subsumed within SPA allocation	329 (63.5)	189 (36.5)		118 (35.9)	131 (39.8)	15 (4.6)	65 (19.8)
Spend more time allocated hours in postgraduate education/supervision	311 (67.5)	150 (32.5)		19 (6.1)	188 (60.5)	63 (20.3)	41 (13.2)
How many hours would you like to spend teaching at all levels			1 (0.2)	11 (2.4)	251 (55.9)	170 (37.9)	16 (3.6)

Number of respondents indicating yes or no. Number of hours per week for respondents was requested where the response to the first question was ‘yes’. PG, postgraduate: SPA, Supporting Professional Activity; UG, undergraduate. nâ€‰=â€‰number of respondents.

**Table 6:** **Medical student and postgraduate trainee allocations**

	Number of students/trainees (%)			
Medical student allocations at any one time	1 to 3	4 to 6	7 to 10	>10
Medical students (n = 375)	261 (69.6)	62 (16.5)	29 (7.7)	23 (6.3)
Postgraduate trainee allocations at any one time	1 to 2	3 to 4	5 to 7	>7
As educational supervisor (nâ€‰=â€‰380)	246 (64.7)	100 (28.5)	34 (8.9)	0 (0)
As clinical supervisor (n = 434)	255 (58.8)	118 (27.3)	34 (7.8)	27 (6.2)

n = number of respondents.

#### Postgraduate

Less than half of respondents reported that they have a set number of hours allocated for postgraduate training specifically in their job plan (Table 5). Of those with teaching within their job plan, approximately a quarter indicated that no hours were specified, half of respondents indicated less than 1 hour per week and only low proportions indicated significant time allocations of 1–3 hours or 3 or more hours per week, respectively (Table 5).

Nearly two-thirds of respondents indicated that their teaching responsibilities formed part of their SPA allocation amongst other non-clinical activities (Table 5). The majority of respondents reported allocations of either less than one hour or 1–3 hours per week, with few indicating time allocations of 3 or more hours per week (Table 5).

Just over two-thirds of respondents reported that they spend more than the allocated hours in education and supervision of postgraduate trainees (Table 5). Nearly two thirds reported spending 1–3 additional hours per week on postgraduate teaching. A fifth of the sample indicated greater time allocations of more than 3 hours per week (Table 5). Approximately two thirds of respondents reported allocations of one or two trainees, with few being allocated more than four (Table 6).

A lower proportion of respondents reported that they spent more than the hours allocated to teaching undergraduate medical students than postgraduate trainees (Table 5). Where additional time was spent in teaching undergraduate medical students, the greatest proportion spent 1–3 additional hours per week (Table 5).

When asked how many hours trainers would like to spend teaching at all levels ideally, the highest proportion favoured 1–3 hours per week, while less than half opted for more than 3 hours per week (Table 5).

### Teaching practice

**Figure 1:**
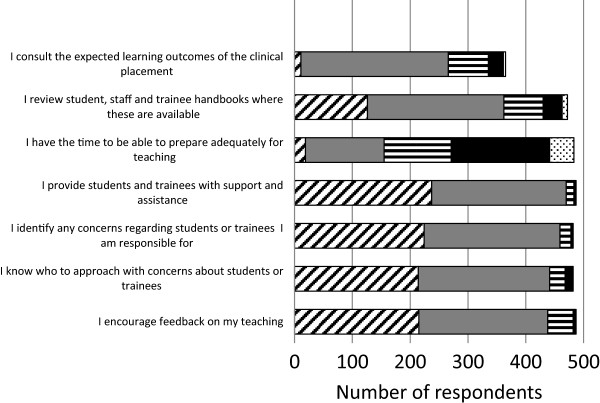
**Teaching practice.** Bars represent number of Likert responses; Strongly agree (diagonally hatched bars), agree (grey bars), Neither agree nor disagree (horizontally hatched bars), disagree (black bars), strongly disagree (stippled bars).

Over three quarters of respondents indicated that they were prepared for their teaching responsibilities (Figure 1) and the substantial majority considered that they delivered their teaching and supervision effectively (Figure 1). There was a much lower level of agreement that sufficient time was available for trainers to prepare adequately for teaching (Figure 1). One respondent commented ‘The preparation for teaching is often done at home in the evenings due to clinical commitments during the day at work. No fixed time [is] allocated for medical student teaching and this is hence done mainly during ward rounds and clinics. No time [is] allocated for medical student appraisal or feedback’.

### Experience of teaching

**Figure 2:**
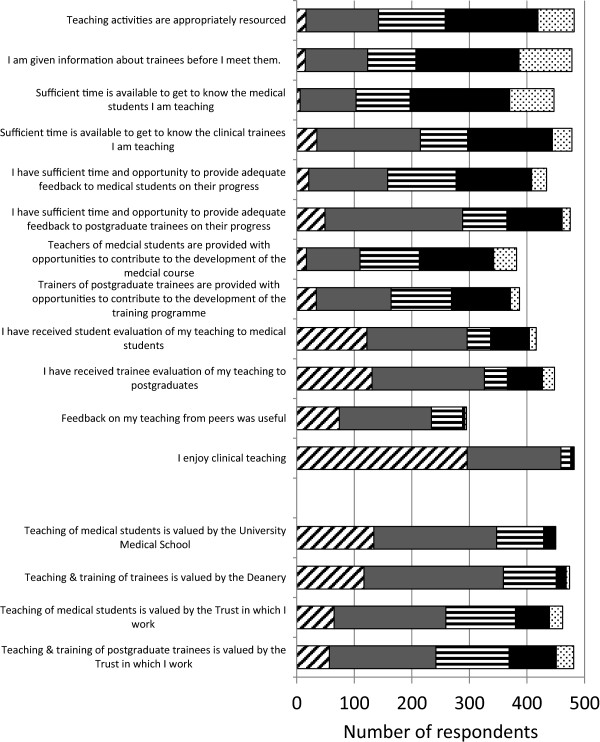
**Experience of clinical teaching.** Bars represent number of Likert responses; Strongly agree (diagonally hatched bars), agree (grey bars), Neither agree nor disagree (horizontally hatched bars), disagree (black bars), strongly disagree (stippled bars).

Levels of satisfaction with the resourcing and time available for clinical teaching/training were relatively low (Figure 2). Just over a quarter of respondents agreed that clinical teaching activities were appropriately resourced. A similar proportion of respondents reported prior receipt of information on their trainees. Similarly, low proportions of respondents reported having sufficient time and opportunity to get to know and provide adequate feedback to medical students (Figure 2). At the postgraduate level, closer to half of respondents had sufficient time to get to know the clinical trainees they were teaching and greater time and opportunities to give feedback to trainees was reported by over half of respondents. Lower proportions of respondents indicated that they had opportunities to contribute to the development of the undergraduate and postgraduate training programmes, respectively (Figure 2). Despite the generally low satisfaction with the support for teaching, the substantial majority of trainers agreed that they enjoy clinical teaching (Figure 2).

### Feedback on teaching

Nearly three quarters of trainers reported receipt of evaluation of their teaching by medical students and postgraduate trainees and there was very high agreement that tutee feedback received was useful (Figure 2). Fewer respondents acknowledged receipt of feedback from informal observation of their teaching or formal observation of their teaching in the last three years, although a majority of trainers agreed or were neutral that feedback on their teaching from peers was useful, with very few disagreeing.

### Esteem for clinical teaching

Three quarters of respondents felt valued by the University Medical School and Deanery for their training of undergraduates and postgraduates, respectively, with a fifth in each case being neutral (Figure 2). Respondents perceived lower value for undergraduate teaching and postgraduate training from their NHS Trust (Figure 2), although esteem varied considerably by individual Trust (not shown).

### Perceived barriers to clinical teaching

**Figure 3:**
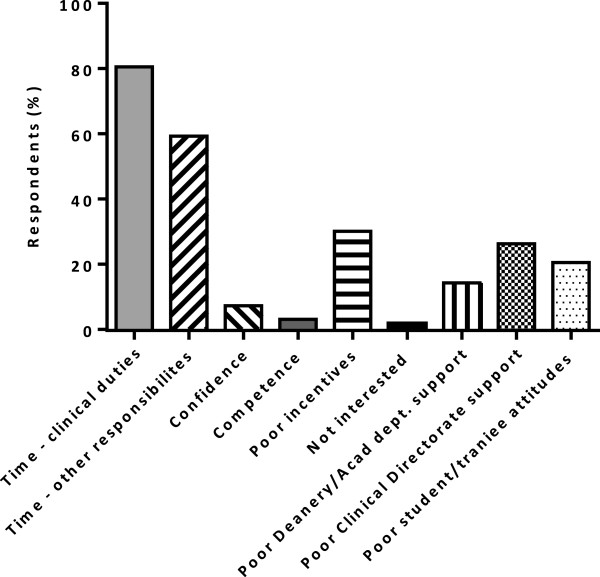
**Barriers to clinical teaching.**

The most significant barriers to teaching were perceived to be the lack of time because of clinical duties and because of other responsibilities (Figure 3). Although fewer than 8% of respondents made free text comments concerning barriers to teaching, the main themes arising were the low time allocation and low prioritisation of teaching over clinical workload, competing priorities within limited SPA time and the need to work out of hours in their own time to complete teaching responsibilities. Lack of incentives, reward or recognition for teaching, poor teaching resources, low confidence in the curriculum and low postgraduate trainee availability due to clinical commitments and poor trainee attitudes were also reported repeatedly, reinforcing the findings of the survey. For example, one respondent commented ‘[The] main problem is in postgraduate education - insufficient time allocated, no formal recognition, pressure of service on us and trainees’. Another indicated ‘very heavy service commitments at DGH [District General Hospital] but I love to teach and have to use personal time for this activity’ and a third reported, ‘No allocated time for being [a] clinical or educational supervisor, only one SPA/week and I use it for other audits, research. I am doing my Supervisor work out of hours’.Lack of incentives for teaching and lack of support from the Clinical Directorate or Deanery/Academic Departments were indicated as other barriers to involvement in teaching (Figure 3). In open comments, the absence of formal recognition or reward for teaching, poor facilities/resources, low time allocation and low prioritisation by Trust managers and poor support from the Deanery were all mentioned repeatedly. Free text comments included ‘Teaching is poorly rewarded other than the satisfaction of doing it. There are multiple other pressures’, ‘[There is a] Lack of recognition by Trust managers of the role of clinical teaching for resources or time and time for being an educational supervisor’ and ‘I get no recognition from the deanery but that does not matter as I get immense recognition from students’. A lower proportion of respondents indicated poor trainee/student attitudes as an additional barrier (Figure 3). Lack of confidence or competence or lack of personal interest were not seen as major barriers to clinical teaching (Figure 3).

A further barrier to teaching was highlighted by some respondents who commented on the lack of availability of trainees for teaching due to the intensity of clinical pressures resulting from inadequate staffing levels. One respondent commented ‘Short-staffed in department so often trainees cannot be released from clinical duties to attend. Trust does not prioritise this over clinical duties’. The difficulty for trainees in accessing trainers appropriately was also mentioned in several responses. For example, ‘Finding convenient times for trainees is becoming increasingly tricky. Trainees have to be quite persistent and anticipate well in advance for meeting’ and ‘trainee availability due to on call/nights significantly reduces the time they can spend with us’. Two respondents commented on the fragmentation of teaching allocation leading to a lack of ‘ownership’ of trainees and the loss of the sense of ‘firm’; ‘Much fragmentation in the medical school rotations. Need stronger “firm” associations rather than current peripatetic style’ and ‘Students are rarely ‘mine’, and yet I end up doing the educating’. A further comment indicated that a lack of opportunity to contribute to the development of the local teaching/training programme was also a disincentive to engagement; ‘No involvement in planning, developing and delivering of local teaching/training programme’.

## Discussion

The main findings of this study were that respondents in the survey like and enjoy clinical teaching despite lack of allocated time, resources and recognition. Most respondents reported teaching at both undergraduate and postgraduate levels, with many working beyond contracted work time to complete their teaching commitments. The majority indicated that they feel confident and competent in their clinical teaching roles. Insufficient dedicated time due to competing clinical service pressures was reported as the major barrier to clinical teaching provision.

The substantial majority of clinical teachers/trainers reported enjoyment of teaching, despite reporting competing heavy demands of clinical service, low time allocations for teaching within job plans, which often compete with other non-clinical activities, poor prioritisation and resourcing for educational activities and poor recognition and value attributed by Hospital Trusts for clinical teaching. Themes identified in this study mirror those identified by undergraduate teachers in the studies of Seabrook [10] and Hendry et al. [11] and suggest that little has changed in the intervening period. There is an apparent mismatch between the expectation of consultants to meet Trust obligations to provide clinical teaching and supervision and an adequate resourcing of consultant time and environment to achieve this. Greater accountability is required at all levels within Trusts to ensure adequate resourcing and recognition of clinical educational activities, to reduce the barriers to clinical education delivery and so safeguard provision of continued high quality clinical education at all levels.

To improve the experiences and engagement of clinical teachers, mechanisms to raise the profile of educational activities within clinical environments should be considered, albeit against the recognition of tightly restricted budgets. Time for educational activity within clinical contracts should be realistic and protected. Clinical educators are often disconnected from curriculum management structures and so care should be taken by curriculum managers to maintain timely updates of appropriate information, to involve tutors in bidirectional quality feedback loops and to offer personal development opportunities. Educational activity should form a significant part of trainer appraisal and mechanisms should be established to ensure regular institutional recognition of provision and excellence in this activity and reward, where this is appropriate. The challenge to clinical education commissioners and providers is to respond to the concerns evidenced by this study to improve the status and quality of clinical educational provision. A key action would be to have clear time allocated to teaching activities within job plans. This should lead to improvements in both the teaching delivered (as teachers could prepare more effectively) and recognition of the value of teaching within the organisation.

### Limitations

This was a web based survey and the response rate was relatively low, although comparable with reported response rates to online surveys [14]. For large populations surveyed, responses from a smaller proportion of the population are required to give confidence in population estimates [15]. Using the guide provided by Dillman [14, 15], the response rate of 25.8% of 2007 individuals surveyed was sufficient for inferences to be drawn from the study data. It is likely that the survey elicited responses from those more interested in teaching, thereby introducing some bias, however, the sample number was relatively large, permitting some generalisation on the practice and experience of clinical teachers. The very high proportion of respondents with teaching responsibilities was suggestive of a bias against non-teaching practitioners in the study. There is a professional expectation on registration in the UK that doctors will contribute to the education and training of other doctors, medical students and non-medical healthcare professionals [1, 16]. Moreover, the majority of consultants’ contracts include time for teaching within their SPA allocation, so the sample studied is likely to be a reasonably accurate representation of the distribution of teachers/non-teachers in the population investigated.

A further limitation of this study was that the teaching practice of clinical educators was surveyed only from the perspective of consultant educators. The study could have been broadened to investigate the contribution made to clinical teaching by postgraduate trainees, both in peer teaching and the teaching of undergraduates. In addition, the perceptions of students and trainees concerning the quality of clinical training provision could have been surveyed to allow triangulation with the views of those responsible for providing the clinical training environment. It is noteworthy, however, that the proportions of students reporting satisfaction overall with their training during the time frame of the survey was high (Leicester Medical School, 90%; Nottingham Medical School, 94%) [17]. Similarly, postgraduate trainee satisfaction in the East Midlands Deanery was generally high (Overall satisfaction, 79.6%; Clinical supervision, 87.8%; Educational supervision, 88.4%; Quality of experience. 80.1%), although Quality of teaching rated somewhat lower (63.4%) and similar to national levels [18]. These findings suggest some disjunction between educator perceptions and student/trainee experience, which is worthy of further study.

### Future studies

Further studies with qualitative methodology could be undertaken to provide increased understanding of the perceptions and experience of clinical educators. Such findings would likely reflect the disparity of local practice and give information as to how localities might improve the experience of their teachers. Of most concern is the major proportion of respondents reporting that they have insufficient dedicated time for clinical teaching due to competing clinical service pressures and/or other responsibilities, raising the question of whether this has a significant detrimental influence on the outcomes of training for students and trainees. The effect of inadequate preparation and low contact time on the quality of student and trainee experience and outcomes is likely to be significant but this is currently unknown. Studies are required to explore the hypotheses that increasing time for educator preparation or student contact time with clinical educators leads to an improvement in student and trainee outcomes and satisfaction. Designing an intervention that could be linked clearly to a predicted outcome without the possibility of confounding interpretations will present a considerable challenge.

## Conclusions

Survey respondents indicated that clinical teaching at undergraduate and postgraduate levels is provided by clinical educators who like and enjoy clinical teaching. Many respondents indicated a need to work beyond contracted teaching time to achieve their teaching commitments and being poorly resourced and recognised for this activity. Increased institutional recognition of teaching contributions and more open involvement of clinical trainers in communications concerning curriculum development and delivery could provide relatively simple and inexpensive enhancements to the engagement and perceived value of clinical educators in secondary care. In addition, attention is required to address the mismatch between expectation and resourcing of clinical teachers.

## Authors’ information

ROBERT I. NORMAN is the Academic Director of the College of Medicine, Biological Sciences and Psychology, University of Leicester, Leicester, UK.

NISHA DOGRA is a Professor of Psychiatry Education at University of Leicester School of Medicine, Leicester, UK.

## Abbreviations

CT: Core Trainee
DGH: District General Hospital
EMSHA: East Midlands Strategic Health Authority
FY1: FY2, Foundation Year 1 and 2
GP: General Practice
NHS: National Health Service
SPA: Supporting Professional Activity
ST: Speciality Trainee
UG: Undergraduate.
